# MicroRNA-132/212 family enhances arteriogenesis after hindlimb ischaemia through modulation of the Ras-MAPK pathway

**DOI:** 10.1111/jcmm.12586

**Published:** 2015-05-06

**Authors:** Zhiyong Lei, Alain van Mil, Maarten M Brandt, Sebastian Grundmann, Imo Hoefer, Michiel Smits, Hamid el Azzouzi, Taro Fukao, Caroline Cheng, Pieter A Doevendans, Joost PG Sluijter

**Affiliations:** a Division Heart and Lungs, Department of Cardiology, University Medical Center UtrechtUtrecht, The Netherlands; b Experimental Cardiology, Erasmus Medical CenterRotterdam, The Netherlands; c Department of Cardiology and Angiology I, University Heart Center Freiburg - Bad KrozingenFreiburg, Germany; d Max Planck Institute of Immunobiology and EpigeneticsFreiburg, Germany; e Division Nephrology & Hypertension, University Medical Center UtrechtUtrecht, The Netherlands; f ICIN, Netherlands Heart InstituteUtrecht, The Netherlands

**Keywords:** miR-132/212, arteriogenesis, Ras-MAPK, hindlimb ischaemia

## Abstract

Arteriogenesis is a complicated process induced by increased local shear-and radial wall-stress, leading to an increase in arterial diameter. This process is enhanced by growth factors secreted by both inflammatory and endothelial cells in response to physical stress. Although therapeutic promotion of arteriogenesis is of great interest for ischaemic diseases, little is known about the modulation of the signalling cascades *via* microRNAs. We observed that miR-132/212 expression was significantly upregulated after occlusion of the femoral artery. miR-132/212 knockout (KO) mice display a slower perfusion recovery after hind-limb ischaemia compared to wildtype (WT) mice. Immunohistochemical analysis demonstrates a clear trend towards smaller collateral arteries in KO mice. Although *Ex vivo* aortic ring assays score similar number of branches in miR-132/212 KO mice compared to WT, it can be stimulated with exogenous miR-132, a dominant member of the miR-132/212 family. Moreover, in *in vitro* pericyte-endothelial co-culture cell assays, overexpression of miR-132 and mir-212 in endothelial cells results in enhanced vascularization, as shown by an increase in tubular structures and junctions. Our results suggested that miR-132/212 may exert their effects by enhancing the Ras-Mitogen-activated protein kinases MAPK signalling pathway through direct inhibition of Rasa1, and Spred1. The miR-132/212 cluster promotes arteriogenesis by modulating Ras-MAPK signalling *via* direct targeting of its inhibitors Rasa1 and Spred1.

## Introduction

Under physiological circumstances, normal adult blood vessels stay quiescent by using various inhibitors to counteract pro-angiogenic signal fluctuation 1. Dysregulation of this balance may cause diseases such as capillary and arterio-venous malformations b. However, under ischaemic conditions, the compensatory growth of blood vessels is an appreciated response, which can be achieved in two ways: by branching from existing vessels (called angiogenesis), or by enlargement of pre-existing collaterals (termed arteriogenesis) 3. The increase in diameter *via* arteriogenesis weights much more than the number of newly formed capillaries *via* angiogenesis and has therefore the potential to become a future therapeutic approach 4 in chronic and acute ischaemic diseases. Many attempts have been made to modulate the pro- and anti-arteriogenic balance 5–7. However, effective therapeutic approaches to promote arteriogenesis are still lacking.

Initial studies have shown an important role for microRNAs (miRNAs) in neovascularization 8–14, but a clear understanding of all players involved is still lacking. It has previously been shown that miR-132 is upregulated in endothelial cells by various pro-angiogenic stimuli such as hypoxia 15, VEGF 10,15, and angiotensin II 16. Overexpression of miR-132 in human umbilical venous endothelial cells (HUVECs) promoted proliferation and migration *in vitro,* and transplanting these cells promoted vascularization *in vivo* 17. In cancer, miR-132 promoted angiogenesis by suppressing one of the GTPase-activating proteins, called RASA1 10. Very low density lipoprotein receptor knockout (KO) mice, displayed an aberrant neovascularization in the retina, associated with increased expression of miR-132. Moreover, inhibition of miR-132 in the retina could reduce aberrant neovascularization 18 or corneal neovascularization 19. Although, the role of this microRNA family was explored for angiogenesis, its influence on hind-limb ischaemia induced arteriogenesis has not been explored. In this study, we combine *in vitro* assays and *in vivo* animal models to explore the role of miR-132/212 in vascular growth during arteriogenesis and to unravel the underlying mechanism.

## Materials and methods

### Generation and genotyping of miR-132/212 KO mice

The generation of miR-132/212 KO mice has been described as previously 20. For genotyping, DNA samples were obtained by ear clipping and used in a GC-Rich PCR kit (Cat. 12140306001; Roche, Switzerland) with the MiR-132/212 primers as shown in the Table S1. PCR products were revealed on a 1% agarose gel: wildtype (WT) genotype shows a predicted band at 1076 bp and the KO genotype at 392 bp.

### Hind-limb ischaemia

This study was approved by the Animal Ethical Experimentation Committee (Utrecht University) and was carried out in accordance with the Guide for the care and use of Laboratory Animals.

Hind-limb ischaemia was applied on 10–12 week old mice [10 WT (C57B6) and 13 miR-132/212 KO] as described previously 21. In brief, mice were anaesthetized with fentanyl (0.05 mg/kg), midazolam (5 mg/kg) and medetomidine (0.5 mg/kg) by intraperitoneal injection and surgical procedures were performed under sterile conditions. A vertical longitudinal incision was made in the right hind-limb and the femoral artery was dissected. To achieve slower recovery, ligation was performed using an electricoagulator at the most proximal position and thereby separating them into two parts. After closure, mice received atipamezole (2.5 mg/kg) and flumazenil (0.5 mg/kg) to recover. Temgesic (0.1 mg/kg) was given every 8 hrs after surgery for 6 times. Measurement of blood flow was performed by scanning both rear paws with an LDI analyzer (Moor Infrared Laser Doppler Imager Instrument, Wilmington, DE, USA), before and after the surgical procedure (days 0, 4, 7, and 14). During the procedure, the animal was kept under 2% isoflurane anaesthesia and its body temperature was strictly maintained between 36.5 and 37.5°C. The images obtained were quantitatively converted into histograms with Moor LDI processing software as described before 22. Data were reported as the ratio of blood flow in the right over left (R/L) hindlimb.

### MicroRNA *in situ* hybridization

The procedure for microRNA *in situ* hybridization has been described previously with slight modification 23. Cryosections were fixed by 4% paraformaldehyde for 10 min., acetylated for 10 min. followed with 10 min. proteinase K treatment (10 μg/ml). Hybridization was performed following manufacturer’s suggestions with DIG labelled miRCURY LNA miRNA detection probes (Exiqon, Vedbaek, Denmark) for miR-132 (38031-15), negative control miR-159 (99003-15) and positive control U6 (99002-15). Sections were subsequently blocked for 1 hr before overnight incubation with anti-DIG alkaline phosphatase antibody (1:1500; Roche, Switzerland). To block endogenous alkaline phosphatase activity, sections were incubated with levamisole solution (DAKO, USA), followed by Liquid Permanent Red (DAKO, USA) incubation for visualization. Blood vessels were stained with lectin BS-1 (1:100; Sigma-Aldrich, USA). Nuclei were stained with Hoechst 33342 (Life Technologies, USA). Images were taken by Zeiss LSM710 and analysed using Zen2012 (Zeiss, Germany).

### RNA isolation and RT-PCR

DNA-free RNA was extracted with Tripure (Roche Applied Science, Switzerland). To perform quantitative PCR (qPCR) for gene expression RNA is transcribed to cDNA using the iScript cDNA Synthesis Kit (Bio-Rad, USA) according to manufacturer’s instructions, and quantitative real-time polymerase chain reaction was performed on a MyIQ single-color qRT-PCR system (Bio-Rad) as described previously 24. All the primers used for qPCR analysis are listed in the Table S1. Mature miR-132 and miR-212 expression levels were measurement by TaqMan® MicroRNA Assay following manufactory’s instruction, using U6 as control.

### Immunofluorescent staining

The following primary antibodies were used: Rasa1 (1:100, clone B4F8, ab2922; Abcam, USA), Spred1 (1:500, ABS186; Millipore, USA), Spry1 (1:500, 13013; Cell Signaling), alpha-smooth muscle actin (αSMA)-FITC Fluorescein isothiocyanate (1:400, F3777; Sigma-Aldrich), followed by secondary antibodies goat anti-mouse and goat anti-rabbit Alexa 555 (1:500; Life Technologies) for detection. In brief, tissues were imbedded in Tissue-Tek® O.C.T™ (SAKURA, Alphen aan den Rijn, The Netherlands) and sectioned to 7 μm thick slices. For the Spred1 and spry1 staining, sections were fixed with cold methanol and subsequently blocked with 10% normal goat serum plus 2% bovine serum albumin (BSA) in TBST Tris-Buffered Saline with 0.1% of Tween 20, containing 0.1% tween 20. Then sections were incubated with primary antibodies diluted in 0.5% BSA in TBST overnight at 4°C. Before incubation with a secondary antibody, slides were washed three times for 10 min. each. For RASA1, sections were first cleared with 1% tween for 30 min., then blocked with affini-pure Fab fragment goat anti-mouse IgG (115-007-003; Jackson Immunoresearch Laboratory, USA) with 10% normal goat serum. Anti-RASA1 antibody was diluted in 0.5% BSA in TBST, applied on the sections overnight at 4°C, followed by biotin-sp-conjugated affini-pure Fab fragment goat anti-mouse IgG(H+L) (1:500, 115-067-003; Jackson Immunoresearch Laboratory) and streptavidin-conjugated Alexa 555. Images were taken by Zeiss LSM700 and analysed using ZEN 2012 software (Zeiss).

### Western blotting

The following primary antibodies were used for western blotting: Rasa1 (1:200, clone B4F8, ab2922; Abcam), Spred1 (1:1000, ABS186; Millipore), Spry1 (1:1000, #13013; Cell Signaling), β-actin (1:15,000; Sigma-Aldrich), p44/42 MAPK(ERK1/2) (1:1000, #9102; Cell Signaling), phosopho-p44/42 MAPK(ERK1/2) (1:1000, #9101; Cell Signaling). Adductor muscles were lysed with EDTA-free lysis buffer (Cat. 04719964001; Roche Applied Science) with 1× protease/phosphatase inhibitor cocktail (#5872; Cell Signaling). Protein concentrations were measured with BCA protein assay kit (23227; Thermo Scientific, USA), separated with NuPAGE bis-tris Precast gels (Life Technologies), and transferred to Polyvinylidene fluoride PVDF membrane with an iblot Western blotting system (Life Technologies), according the manufacturer’s instructions. Membranes were first blocked with 5% blotting grade blocker (#170-6404; Bio-Rad) with exception of the detection of phosoph-ERK1/2 in which 5% BSA was used. After washing, Horseradish Peroxidase HRP-conjugated secondary antibody was used for enhanced chemiluminescence ECL detection (Sigma-Aldrich, USA).

### Aortic ring assay

Aortas from both WT and miR-132/212 KO mice were surgically isolated, cleaned, dissected into 0.5 mm segments and embedded into fibrin as described before 25. For rescue, aortic ring segments were transfected overnight either with 50 nmol/l microRNA mimics as indicated by siPORT NeoFX prior to embedding. 25 ng/ml recombinant mouse vascular endothelial cell growth factor (VEGF164; 493-MV-005; R&D, USA) was added and replaced on day 4. Pictures were taken on day 7 and the number of branches were counted under an inverted microscope.

### Cell culture and transfection

Human umbilical venous endothelial cells (Lonza, Breda, the Netherland) were cultured in EGM2 according to manufacturer’s instructions, and all experiments were performed before passage 7. HUVECS were transfected with either 20 nmol/l Spred1 (s46287), Spry1 (s20026), Rasa1 (120290), Silencer select negative control#1 (4390843), or with mirVana miRNA mimic negative control (4464085), hsa-miR-132-3p mimics (MC10166), hsa-miR-212-3p mimics (MC10340), mirVana miRNA inhibitor negative control1 (4464077), hsa-miR-132-3p inhibitor (AM10166), hsa-miR-212-3p inhibitor (AM10340; all from Life Technologies) using Lipofectamine 2000 (Life Technologies).

### 3′-untranslated region reporter generation and luciferase assay

A 1 kb fragment, which flanks conserved miR-132-binding sequences of the *spred1* untranslated region (UTR), and the full-length *Spry1* 3′UTR were cloned into the pMIR-REPORT Luciferase vector (Ambion, USA), as described previously 23. Mutations in the seed-region were generated by Q5 Site-Directed Mutagenesis kit (New England Biolabs, USA). All the primers used for cloning and mutagenesis are listed in the Table S1. To determine suppression efficiency of miR-132 and 212 on these targets, HEK293 cells were co-transfected with 200 ng of pMIR-REPORT- 3′UTR Luciferase vectors, or one of the mutated vectors, and a pMIR-REPORTβ-gal control plasmid to normalize for transfection efficiency. In addition, 25 nmol/l miR mimic controls, miR-132 mimics or miR-212 mimics were introduced by using Lipofectamine 2000 (Life Technologies). Luciferase and β-galactosidase activity was assessed after 48 hrs with the Luciferase Assay System and β-galactosidase Enzyme Assay System (both from Promega, USA), respectively, as previously described 24.

### *In vitro* angiogenesis assay

Human umbilical vein endothelial cells (Lonza) and human brain vascular pericytes (#1200; Sciencell, San Diego, USA) were cultured on gelatin-coated plates in EGM2 medium (EBM2 medium supplemented with EGM2 bullet kit and 2% FCS; Lonza) and DMEM (10% FCS; Lonza), respectively, in 5% CO_2_ at 37°C. Lentiviral transfected HUVECs expressing green fluorescent protein (GFP) and pericytes were used at passage 6–8. miR-132 and miR-212 were inhibited or enhanced in HUVECs only, either by using anti-miR-132 and anti-miR-212, or by supplementing miR-132 mimics and miR-212 mimics, respectively. Control cells were transfected with non-targeting miR and anti-miR controls. To monitor the effects of miR-132 and miR-212 in angiogenesis, transfected HUVEC-GFP and PKH26 stained pericytes were suspended in a 2.5 mg/ml collagen type I (BD Biosciences, USA) as described by Stratman *et al*. 26. Co-cultures were imaged after 96 hrs incubation in 5% CO_2_ at 37°C by fluorescence microscopy, thereby acquiring four planes of images, followed by 3D-analysis using a commercial analysis system (Angiosys, Buckingham, UK).

### Phospho-ERK1/2 Bio-PlexPro™ assay

In brief, 48 hrs after transfection of indicated siRNAs or microRNA mimics, EGM2 was removed and replaced with EBM2 for 3 hrs to starve the HUVECs. Subsequently, 25 ng/ml recombinant human VEGF165 (293-E-010; R&D) was added and cells were harvested at indicated time points and lysed with EDTA-free lysis buffer (Cat. 04719964001; Roche Applied Science) with 1× protease/phosphatase inhibitor cocktail (#5872; Cell Signaling). Protein concentrations were measured with the BCA protein assay kit (23227; Thermo Scientific) and diluted into 200 μg/ml. Phospho-ERK1/2 Bio-PlexPro™ assay was performed according to the manufacture’s instruction. Of each sample, 100 μl was incubated with capture antibodies (171-v50006M, 171-v60003M; Bio-Rad), and after washing, streptavidin-PE was applied for visualization. Samples were processed with the Bio-plex 200 (Bio-Rad) and data were analysed with Bio-Plex Data Pro software (Bio-Rad) and Graphpad Prism 6.0.

### Statistical analysis

Data were using Graphpad Prism 6 and comparisons were performed with *t*-test or paired *t*-test between two groups, and anova for multiple comparisons. Data were presented as mean ± SEM. *P*-values are indicated as follows: *P < 0.05; **P < 0.01; ***P < 0.001, *P* < 0.05 is considered as significant.

## Results

### MiRNA-132 and miR-212 is upregulated upon hind-limb ischaemia

**Figure 1:**
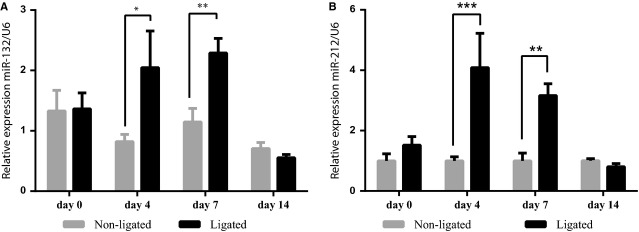
miR-132/212 expression after hind-limb ischaemia. (A) miR-132 expression as measured by qPCR assays. *N* = 5, values in the graph are shown as mean ± SEM, *P < 0.05; **P < 0.01. (B) miR-212 expression as measured by qPCR assays. *N* = 5, values in the graph are shown as mean ± SEM, **P < 0.01; ***P < 0.001.

To understand the function of miR-132 and miR-212 in arteriogenesis, we performed hind-limb ischaemia on WT mice and checked the expression of these two microRNAs in the thigh muscle at different time points after hind-limb ischaemia. By qRT-PCR, we found that miR-132 and miR-212 levels were significantly increased on day 4 and day 7 (Fig.1A and B) after hindlimb ischaemia in the adductor muscle.

The miR-132/212 locus is positioned in the first intron of the human 1700016p03 gene, whose function remains to be investigated. miR-132 and 212 are transcribed as a single transcript and further processed into two mature microRNAs, which are highly conserved among different species (Fig. S1A). Although these two miRNAs share the same seed sequences and hereby belong to the same miRNA family, the level of mature miR-132 expression is significantly higher than that of miR-212 in the thigh muscle, indicating that miR-132 might be more active in the arteriogenic response, as previously reported for miR-212 being a more dominant miRNA in angiogenesis (Fig.1A).

To further understand its function, we analysed which cell types express miR-132 in hind-limb tissue by *in situ* hybridization. As expected, we found that miR-132 is expressed in endothelial cells (lectin BS-1 positive cells) of blood vessels and in cells surrounding the endothelial cell layer in WT mice only (Fig. S1B).

### MicroRNA-132/212 is involved in arteriogenesis after hindlimb ischaemia

**Figure 2:**
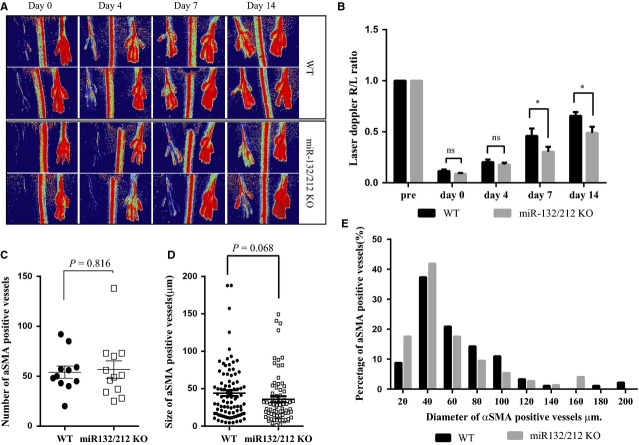
miR-132/212 knockout mice show slower blood flow recovery rate after hindlimb ischaemia. (A) Laser Doppler images of WT and miR-132/212 KO at 0, 4, 7 and 14 days after ligation of femoral artery. Low or no perfusion is displayed as dark blue, whereas the highest degree of perfusion is displayed as red. (B) Quantification of laser Doppler image (Ratio R/L) as shown in A (*N* = 10 for WT and 13 for KO. Values in the graph are shown as mean ± SEM, *P < 0.05. (C) Quantification of number of αSMA positive vessels as determined by αSMA staining on adductor muscle on day 14. (*N* = 10 for WT and 13 for KO. Values in the graph are shown as mean ± SEM). (D) Quantification of the diameter of αSMA positive vessels as determined by αSMA staining on adductor muscle on day 14 (*N* = 10 for WT and 13 for KO. Values in the graph are shown as mean ± SEM). (E) Quantitative analysis the percentage of arteries in different size range. Note the higher percentage in the small vessels (≦400 a.u.) but lower in the larger vessel in the miR-132/212 KO mice (*N* = 10 for WT and 13 for KO.

The increased expression upon hindlimb ischaemia and the vascular localization of miR-132 suggests that miR-132 may play a role in vascular growth, for example, in arteriogenesis. To test this, we compared the arteriogenic response between WT and KO mice. Blood flow perfusion ratio in the miR-132/212 KO mice was significantly lower compared to their WT litters at day 7 and 14 as measured by laser Doppler (Fig.2A and B), indicating a slower perfusion restoration. The total number of αSMA positive vessels in the adductor muscle was similar between WT and KO mice (Fig.2C), but there is a clear trend towards smaller collateral arteries in KO mice, as determined by the cross-sectional diameter of αSMA-positive arteries (Fig.2D and E).

### miR-132/212 promotes endothelial cells neovascularization responses *in vitro*

To further investigate the effect of microRNA-132/212 on vascular growth, we modulated miR-132/212 activity with overexpression or inhibition approaches in different *in vitro* neovascularization assays.

Firstly, we performed WT and miR-132/212 KO mice-derived aortic ring assay 25. In the growth factor rich environment, we observed a slight decrease in the number of branches in aortic rings from KO mice compared to WT control. Interestingly, transfection of miR-132 mimics rescued, and even significantly enhanced activation *via* increasing vascular branching. Although similar effect were observed with miR-212, the effects were less pronounced as compared with miR-132 (Fig. S1C and D).

**Figure 3:**
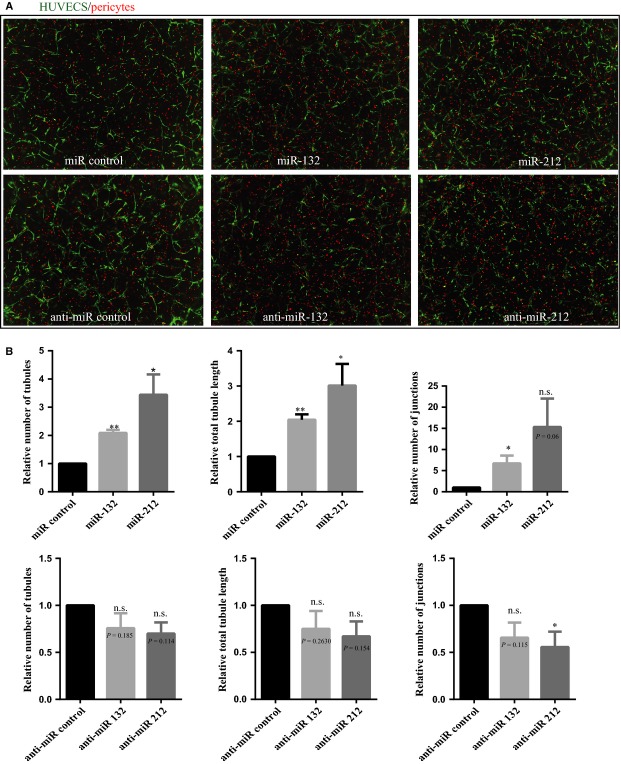
Effect of miR132 and miR212 in HUVECs angiogenesis in co-culture with pericytes. (A) Representative image from HUVECs and pericytes co-culture assay with miR-132 and 212 transfection. HUVECs labelled in green with GFP, pericytes in labelled with PKH26 in red. (B) Quantification of the HUVECs and pericytes co-culture assay with anti-miR-132 and 212 transfection (*N* = 3, values in the graph are shown as mean ± SEM, *P < 0.05; **P < 0.01).

Secondly, we performed a HUVEC and pericyte co-culture assay, thereby better mimicking the *in vivo* situation by taking the interplay between endothelial cells and pericytes into account 26. As shown in Figure3A, supplementing miR-132 and miR-212 mimics to HUVECs enhanced the total number of junctions, tubules and tubule length compared to that of miR controls. Conversely, inhibiting miR-132 and miR-212 using anti-miRs resulted in some decline in the total number of junctions, tubules and tubule length (Fig.3B).

### *Spred-1*, *Spry1* and *Rasa1* are direct targets of the microRNA-132/212 family

**Figure 4:**
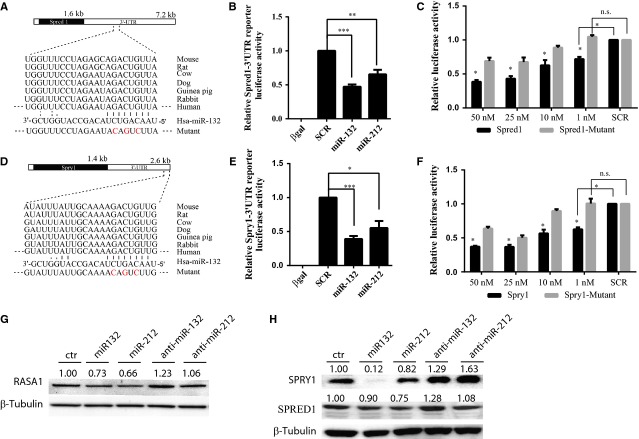
Identification of*Spred1* and *Spry1* as direct miR-132/212 targets by luciferase assay and in cultured HUVECs. (A) The position of predicted miR-132 and miR212 targets of Spred1 by Targetscan and mutant form of 3′UTR as indicated in red. (B) Luciferase assay of *Spred1*-3′UTR reporter in response to transfection with indicated scramble or microRNA mimics at final concentration of 25 nM. N ≥ 3, values in the graph are shown as mean ± SEM, **P < 0.01; ***P < 0.001. (C) Luciferase activity of wildtype and mutant *Spred1*-3′UTR in response to different dose of miR-132, *N* = 6 for WT; *N* = 3 for mutant. Values in the graph are shown as mean ± SEM *P < 0.05. (D) The position of predicted miR-132 and miR212 targets of Spred1 by Targetscan and mutant form of 3′UTR as indicated in red. (E) Luciferase assay of Spred1-3′UTR reporter in response to transfection with indicated scramble or microRNA mimics at final concentration of 25 nM. N ≥ 3, values in the graph are shown as mean ± SEM; **P < 0.05; ***P < 0.001. (F) Luciferase activity of wildtype and mutant Spred1-3′UTR in response to different final concentration of miR-132 mimics transfection, *N* = 6 for WT; *N* = 3 for mutants. Values in the graph are shown as mean ± SEM, *P < 0.05. (G) RASA1 expression after miR-132, miR212 overexpression and inhibition in HUVECs. The number above indicates the relative expression compared with sham normalized by β-Tubulin expression. (H) SPRED1 and SPRY1 expressions after miR-132, miR212 overexpression and inhibition in HUVECs. The number above indicates the relative expression compared with sham normalized by β-Tubulin expression.

Based on Ago-Hits-clip 27, PAR-CLIP 28 and CLASH studies 29 and our results above, we decided to focus on targets related to growth factor signalling. In combination with bioinformatics target site prediction algorithms (Targetscan), we selected *Rasa1*, *Spred1* and *Spry1* which have a high prediction context score and are conserved among species, as shown in Figure4A and D. Since *Rasa1* was already a confirmed miR-132 target 10, we only cloned the 3′UTR of *Spred1* and *Spry1* into a luciferase reporter vector and analysed whether miR-132 and miR-212 could suppress luciferase activity in HEK293 cells. We found that both miR-132 and miR-212 can significantly suppress the *Spred1*-3′UTR and *Spry1*-3′UTR luciferase activity at 25 nmol/l, compared with scramble control miRNAs (Fig.4B and E). Additionally, we performed a dose-response assay with *Spred1*-3′UTR, *Spry1*-3′UTR and 3′UTRs with three mutated nucleotides in indicated binding regions (Fig.4A and D). Inhibitory effects were dose-dependent and mutations within the seed region significantly affected the suppressing effects on luciferase activity, even completely abolishing the suppressive effect at a concentration of 1 nmol/l (Fig.4C and F).

We next investigated the direct regulation of these targets in HUVEC cells. Following overexpression of miR-132 and miR-212 in HUVECs in the co-culture assays, we detected reduced levels of Spred1, Spry1 and Rasa1 protein; while inhibition of the miR-132 and miR-212 led to elevated Spred1, Spry1 and Rasa1 expression. This indicates that these genes are also regulated by miR-132/212 in HUVECs (Fig.4G and H).

### miR-132/212 modulates growth factor-activated Ras-MAPK signalling in HUVECs

**Figure 5:**
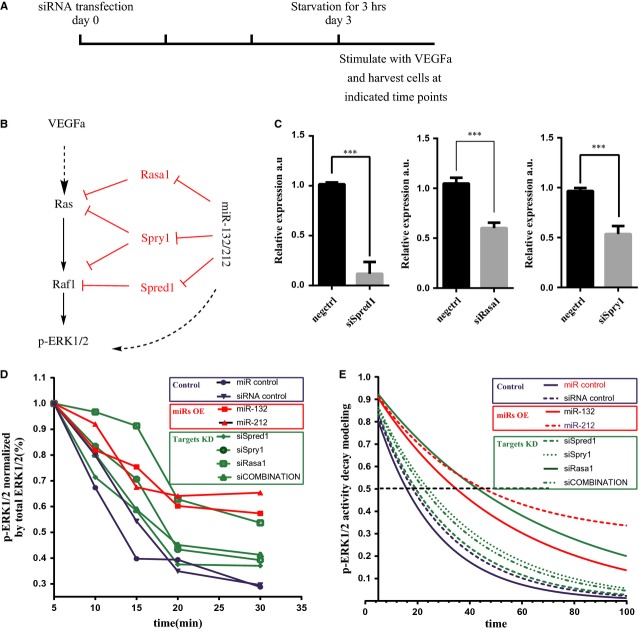
miR-132/212 modulate Ras-MAPK signalling by suppressing Rasa1, Spred1 and Spry1 in HUVECs. (A) Experimental setting for quantitative measure of active ERK1/2 using Bio-plex phosopho-ERK1/2 assay. (B) A working model for miR-132/212 in modulation of Ras-MAPK pathway. (C) Quantification of Spred1, Spry1 or Rasa1 expression level after siRNA transfection against Spred1, Spry1 or Rasa1 in HUVECs, Values in the graph are shown as mean ± SEM, *** *P* < 0.001 *n* = 3. (D) Quantification of phosophorylated ERK1/2 level by Bio-plex pro phosopho-ERK1/2 set. Note the sustain ERK1/2 phosphorylation is prolonged after miR-132, 212 transfection or siRNA against Spred1, Spry1, Rasa1 or combinations of the three. (E) Modelling the decay of phosophorylated ERK1/2 level from D.

Spred1, Spry1 and Rasa1 are known inhibitors of Ras-MAPK signalling and their inhibition can prolong Ras-MAPK signalling upon growth factor stimulation 30–33. We therefore tested whether miR-132/212 could prolong Ras-MAPK signalling by inhibiting *Spred1*, *Spry1* and *Rasa1* in HUVECs. Compared with siRNA controls and miR controls, overexpression of miR-132 and miR-212 or knockdown of *Spred1*, *Spry1*and *Rasa1*, indeed prolonged ERK1/2 phosphorylation (Fig.5A–D). By using a non-linear one-phase exponential decay model and interpolation of the time of phosphorylated ERK1/2T_1/2_ to reach 50%, we observed that T_1/2_ was prolonged both by overexpression of miR-132/212 and by knockdown of its targets (Fig.5E).

### *Spred1*, *Spry1* and *Rasa1* knockdown promotes endothelial cells neovascularization responses *in vitro*

**Figure 6:**
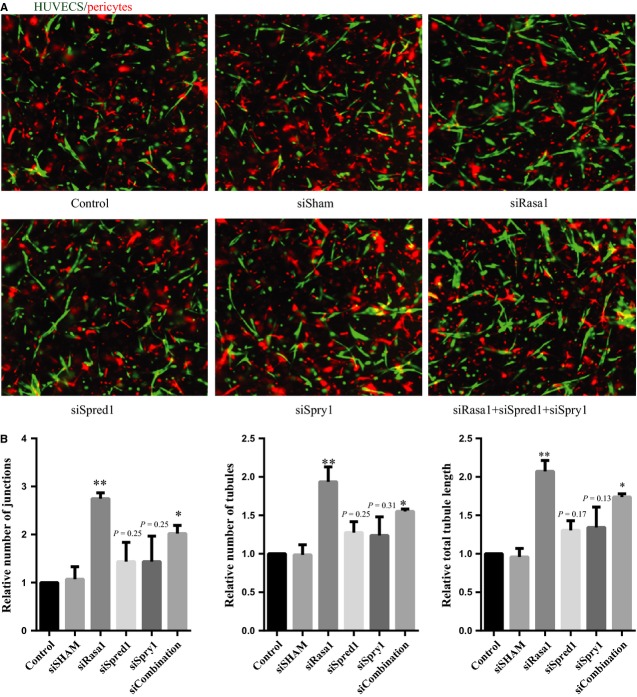
Knockdown targets of miR132 and miR212 Rasa1, Spred1 and Spry1 mimics effect of miR-132 and miR-212 in HUVECs pericytes neovascularization assay. (A) Representative image from HUVECs and pericytes co-culture assay after transfection with siRNAs against Spred1, Spry1, Rasa1 and combination of these three siRNA. (B) Quantification of the number of tubules, junctions and total tubule length in the HUVECs and pericytes co-culture assay after transfection with siRNAs against Spred1, Spry1 and Rasa1, Values in the graph are shown as mean ± SEM, *P < 0.05; **P < 0.01; *n* = 3.

We have shown that overexpression of miR-132 or miR-212 promotes endothelial cells neovascularization. As microRNA functions by inhibiting its targets, we reason that knockdown of *Spred1*, *Spry1* and *Rasa1* should have similar effect as overexpression of miR132 and miR-212. As expected, subsequent knockdown of *Spred1*, *Spry1*, *Rasa1* and a combination of these three *via* siRNA knockdown in HUVECs (Fig.6C) showed similar neovascularization responses as overexpression of miR-132 or miR-212, total number of junctions, tubules and tubule length were increased compared to control conditions (Fig.6A and B).

### miR-132/212 family modulates Ras-MAPK signalling by targeting *Spred1*, *Spry1* and *Rasa1 in vivo*

**Figure 7:**
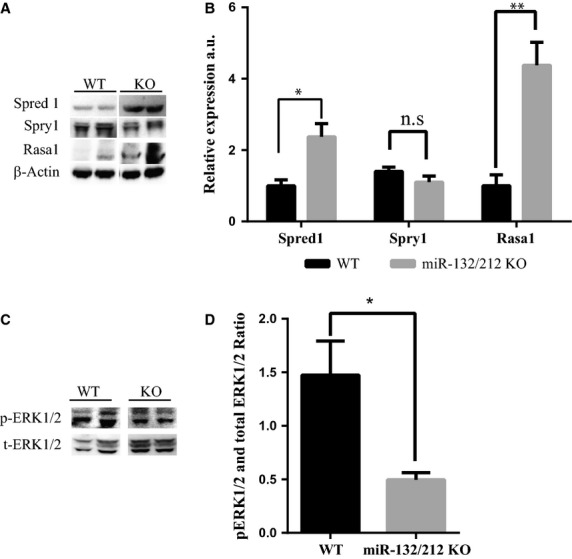
Expression of miR-132/212 targets and phosophorylated ERK1/2 in ischaemia limb. (A) Spred1, Rasa1 and Spry1 expression as determined by Western blot, normalized by β-Actin. (B) Quantification Spred1, Rasa1 and Spry1 expression as determined by Western blot in B. (C) Phosophorylated ERK1/2 expression in the thigh WT and KO mice on 14 days after hindlimb ischaemia as determined by Western blot on day 14. (D) Quantification of phosophorylated ERK1/2 expression in the thigh WT and KO mice on 14 days after hindlimb ischaemia as determined by Western blot, normalized by β-Actin.

We reasoned that if *Spred1*, *Spry1* and *Rasa1* are *in vivo* targets of miR-132 or 212, they should be expressed in the arteries of the thigh muscle. By immunofluorescent staining, we observed that *Spred1*, *Spry1* and *Rasa1* could all be detected in the vascular wall (Fig. S2). By comparing the expression of *Spred1*, *Spry1* and *Rasa1* in the adductor muscle from WT and KO after femoral artery ligation using Western blot, we observed that Spred1 and Rasa1 are significantly more present in the KO mice (Fig.7A and B). Surprisingly we found that there is no difference in the Spry1 protein between WT and KO mice (Fig.7A and B). Next we asked if higher level of Spred1 and Rasa1 expression could have an effect on the Ras-MAPK pathway. By Western blotting we detected lower phosphorylated ERK1/2 in the area of blood vessel growth in KO mice 14 days after hindlimb ischaemia (Fig.7C and D).

## Discussion

Here, we show that upregulation of miR-132 and miR-212 upon hindlimb ischaemia is involved in the arteriogenic response: microRNA-132/212 KO animals display delayed perfusion restoration upon femoral artery occlusion. Furthermore, we demonstrate that this effect is attributable to miR-132/212 modulation of the Ras-MAPK signalling pathway through direct targeting of *Spred1* and *Rasa1*. To the best of our knowledge, this is the first study showing a single microRNA family, and probably mediated *via* the more abundantly expressed miR-132, that can facilitate the arteriogenic responses by suppressing multiple targets within the Ras-MAPK pathway.

The Ras-MAPK pathway is very important in neovascularization during development and after ischaemic challenges. In the developing retina, for example, this physiological pathway only becomes apparent in active sprouting endothelial cells 18. Reduced ERK1/2 activation leads to reduced lumen formation, whereas excessive activation of ERK1/2 results in larger arteries 34,35. Upon ischaemic challenge, *e.g*. in hindlimb ischaemia models, shear stress-stimulated endothelial cells induce Monocyte Chemotactic Protein 1(*MCP-1*) expression, which in turn attracts neutrophil granulocytes and macrophages 36. These circulating inflammatory cells start producing growth factors which eventually activate Ras-MAPK signalling pathway in the smooth muscle cells and endothelial cells further and promote their proliferation and extracellular matrix remodelling. Recently, attempts have been made to interfere in arteriogenesis through manipulation of Ras-MAPK signalling genetically and chemically, for example by suppression of Sproutys to promote blood flow recovery in the hindlimb ischaemia model 7. Since therapeutic activation of Ras-MAPK signalling is still challenging, inhibition of their endogenous inhibitors using microRNA therapeutics holds a great promise as an alternative strategy.

We confirmed that *Rasa1* is a direct target of miR-132 and miR-212, and further expanded their target spectrum thereby including *Spred1* and *Spry1*. Using 3′UTR reporters of *Spred1* and *Spry1*, we demonstrated a direct binding of miR-132 and 212, which was abolished by disrupting the corresponding binding sites. Knockdown of these three targets mimicked overexpression of miR-132 or miR-212 in the *in vitro* neovascularization assay and on the modulation of phosphorylated ERK1/2. Our data show that Rasa1 is the most potent regulator among the three targets in promoting neovascularization and prolonging pERK1/2 activation in HUVECs, probably as being the most upstream signalling molecule as compared to Spry1 and Spred1. Another possibility is genetic redundancy, in which the loss of *Spred1* or *Spry1* can be compromised partially by other family members; Spry1 has at least four homologues 37 and Spred1 has at least two homologues in mice 38. Our *in vitro* observations were confirmed *in vivo* where levels of Rasa1 and Spred1 were significantly higher in the adductor muscle in the miR-132/212 KO mice upon hind-limb ischaemia. Although Spred1 and Rasa1 protein levels were higher in miR-132/212 KO mice, Spry1 expression levels were similar between WT and KO mice. Accordingly, we demonstrated lower levels of phosphorylated ERK1/2 as a downstream effect of lower active Ras-MAPK signalling.

The biological function of miR-132 and miR-212 may be different, although they share the same seed sequence. It has been shown that microRNA targets determination is beyond the seed sequence 39. Consistent with this notion, we observe different effects in various assays. Since both miR-132 and miR-212 are removed in the KO mice, it is impossible to determine which one should be responsible for the impaired arteriogenesis response. Given the fact that the expression of the mature miR-132 is 40-fold higher than miR-212 (Fig. S1A), we tend to believe that miR-132 plays a major role in the arteriogenic response after hindlimb ischaemia. However, it is still possible that a specific cell population, highly expressing miR-212 but not miR-132, is more important for the vascular growth after hindlimb ischaemia. In line with this hypothesis, a recent study showed that miR-212 is stronger in the regulation of vasodilatation than miR-132 40. To exclusively clarify the different roles and locations of these two microRNAs, improved microRNA *in situ* techniques with higher sensitivity are needed that can detect low abundant expressed microRNA in combination with mice with individual microRNA KOs. Our results demonstrate a new role for miR-132 and miR-212 in the facilitation of the arteriogenic responses after hind-limb induced by targeting and enhancing Ras-MAPK signalling. This extends the role for miR-132 beyond the ischaemic challenges and promoting angiogenesis. It would be interesting to test if we can enhance arteriogenesis by specifically deliver these two microRNAs in the ischaemia vasculature or in combination with other Ras-MAPK activators such as growth factors to further boost their pro-arteriogenic capacity. However, local delivery strategies should still be further improved 41.

## Supporting Information

**Figure S1.** The expression of the miR-132/212 and their roles in Ex vivo neovascularization assay. **Figure S2.** The expression of miR-132/212 targets Spred1, Spry1 and Rasa1 in wildtype thigh blood vessels characterized by immunofluorescent staining. **Table S1.** Primers and oligos nucleotides used in this study.

** **

** **
