# Supplementary material for: MicroRNA-132/212 family enhances arteriogenesis after hindlimb ischaemia through modulation of the Ras-MAPK pathway
Source: J Cell Mol Med. 2015 May 6;19(8):1994–2005. doi: 10.1111/jcmm.12586 (PMC4549050; doi:10.1111/jcmm.12586)
Supplement: Supplementary file 1 [file jcmm0019-1994-sd1.docx]

Online Supplemental Material

*Online Supplementary figures and tables*

**Supplementary Figure 1.** The expression of the miR-132/212 and their roles in Ex vivo neovascularization assay.

1. qPCR analysis the expression of miR-132 and 212 in thigh muscle. Values in the graph are shown as mean+-SEM, **p<0.01; n=3
2. miR-132 expression in the endothelial cells in the thigh muscle blood vessel as detected by miR-132 in situ, miR-132 in red, endothelial cells in green, nuclei in blue, bar=5μm.
3. Representative pictures from aorta ring assays. bar=200µM
4. Quantification of aorta ring branching from WT, KO, and KO transfected with miR-132 and 212 precursors (n>3, Values in the graph are shown as mean ± SEM, **p<0.01).

**Supplementary Figure 2.**The expression of miR-132/212 targets Spred1, Spry1 and Rasa1 in wildtype thigh blood vessels characterized by immunofluorescent staining. Note the overlapping expression of Spred1 (in A), Rasa1(in B) with αSMA. Spry1 are expressed in the interior layer CD31 positive cells layers in the vessel as shown in C with white arrow and also expressed in the cells which are either CD31 or αSMA positive as indicated by red arrow. Spred1, Rasa1 and Spry1 in red, αSMA in green, CD31 in purple, nuclear was stained with DAPI in blue. Bar=10mm

**Supplementary Table 1.** Primers and oligos nucleotides used in this study.

**Supplementary Table 1.** Used primers and oligos nucleotide sequences .

| **Primer name** | **Sequence(5’-3’)** | **Application** |
| --- | --- | --- |
| Mir132-KO-GT-P1 | ATCCTTTCAAGAAAGTGGGGAGA | genotyping |
| Mir132-KO-GT-P2 | TCTGAGGAGGATGTTCAGACAC | genotyping |
| hSpred-utr_SDM1_F | TCCTAGAATAcagtcTTAACTTTCAAAATTTTTATTGGTG | mutagenesis |
| hSpred-utr_SDM1_R | AACCAAGTTTAGCTGAAAAC | mutagenesis |
| hSpry1utr_SDM1_F | TATTGCAAAAcagtcTTGAAATGTACTCATGTTTG | mutagenesis |
| hSpry1utr_SDM1_R | AATACCATAAAATAATAGAGATTCG | mutagenesis |
| hSpred1-3UTR_F1 | ATCGGCTAGCAAGAAGAGGGTATTTCTA | cloning |
| hSpred1-3UTR_R1 | ATCGAAGCTTATACACATGTTTAAGAAT | cloning |
| hSpry1-3'UTR_F1 | ATCGGCTAGCGGAGGTGGGTTGTACCTC | cloning |
| hSpry1-3'UTR_R1 | ATCGAAGCTTGACAGTTTCATTAATTAA | cloning |
| hSpry1_F1 | GCAGTGGCAGTTCGTTAGTTG | qPCR |
| hSpry1_R1 | CAGTAGGCTGAATCTCTCTCTCA | qPCR |
| hSpred1_F1 | AAGGATGCCCCGAATCAAAAA | qPCR |
| hSpred1_R1 | GGCTTGGCTTTGCATGTAGAC | qPCR |
| hRASA1_F1 | GGGAGCTGGACTGACAGG | qPCR |
| hRASA1_R1 | AGAGTCTCAGCAAGCAACGA | qPCR |
| mACTB_F1 | GGCTGTATTCCCCTCCATCG | qPCR |
| mACTB_R1 | CCAGTTGGTAACAATGCCATGT | qPCR |
| Gapdh_F2 | GGCATGGACTGTGGTCATGA | qPCR |
| Gapdh_R2 | TTCACCACCATGGAGAAGGC | qPCR |
| mSpry1_F1 | ATGGATTCCCCAAGTCAGCAT | qPCR |
| mSpry1_R1 | CCTGTCATAGTCTAACCTCTGCC | qPCR |
| mSpred1_F1 | GAGATGACTCAAGTGGTGGATG | qPCR |
| mSpred1_R1 | TCTGAAAGGTAAGGCCAAACTTC | qPCR |
| mRasa1_F1 | GTGGTGTACAGCAGCACGTA | qPCR |
| mRasa1_R1 | ATTACCACGGTGGGCATACT | qPCR |
