# Supplementary figures and images for: MicroRNA-132/212 family enhances arteriogenesis after hindlimb ischaemia through modulation of the Ras-MAPK pathway
Source: J Cell Mol Med. 2015 May 6;19(8):1994–2005. doi: 10.1111/jcmm.12586 (PMC4549050; doi:10.1111/jcmm.12586)

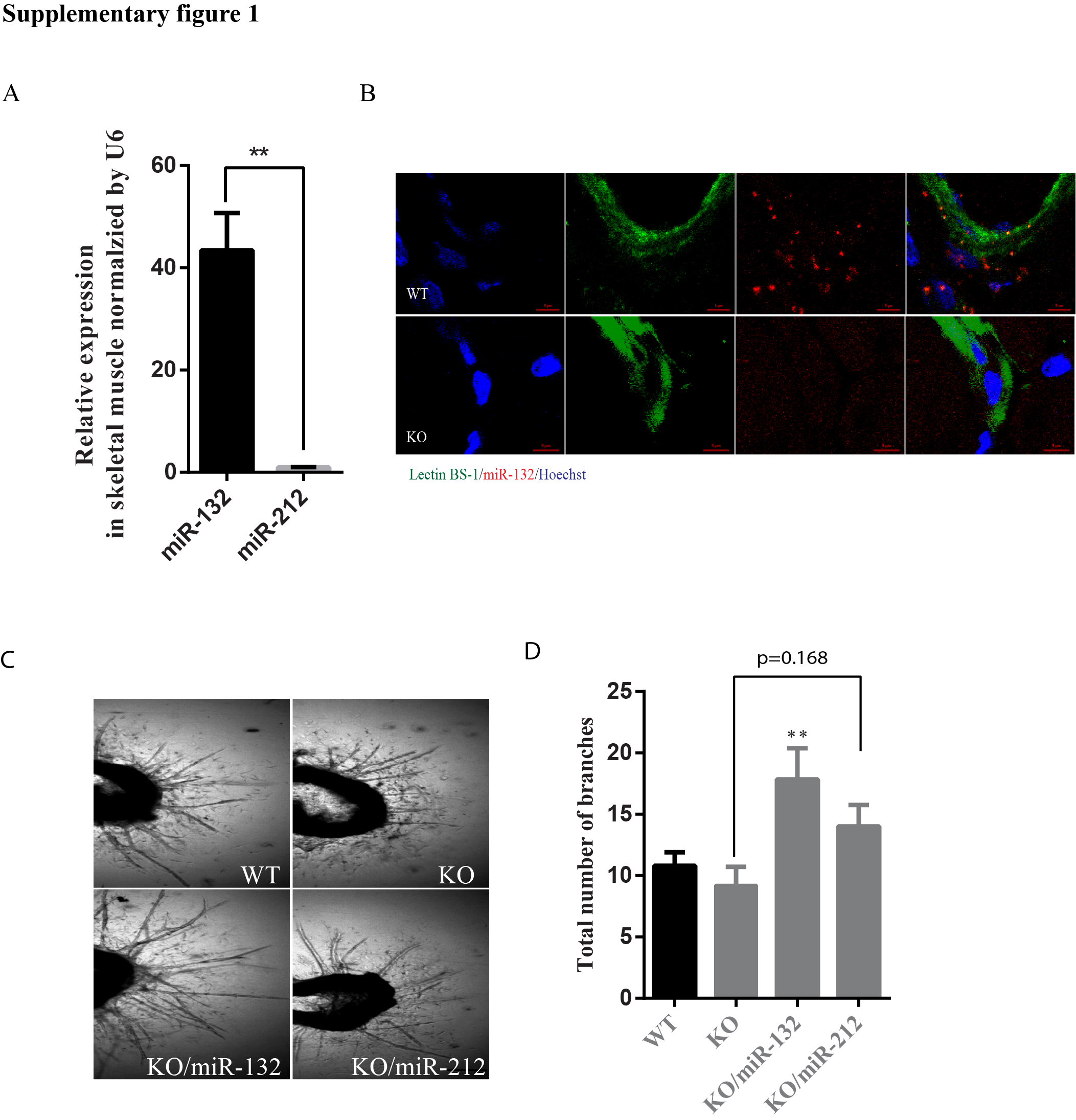

Supplement: Supplementary file 2 [file jcmm0019-1994-sd2.tif]

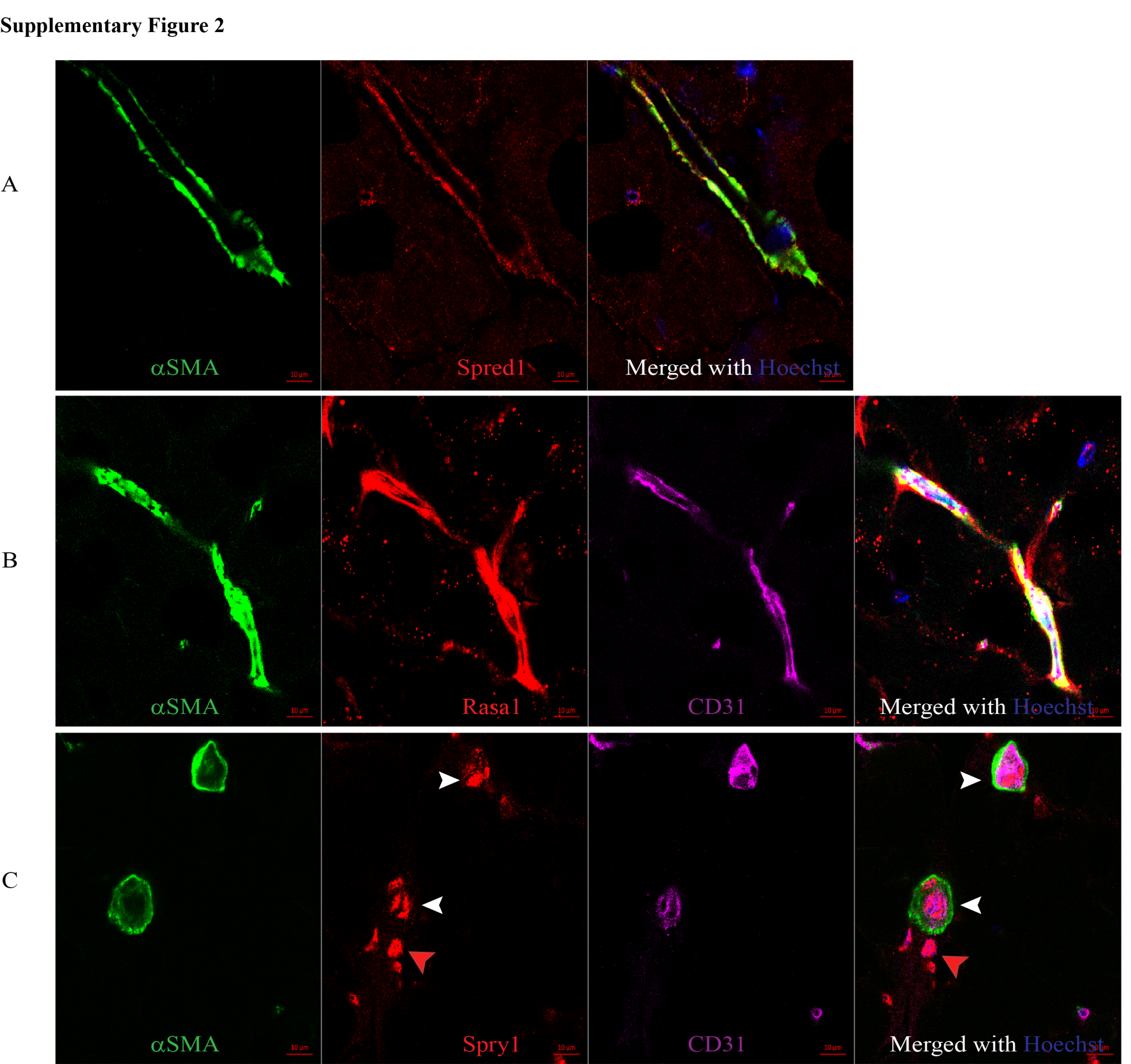

Supplement: Supplementary file 3 [file jcmm0019-1994-sd3.tif]
